# Ursolic acid alleviates airway-vessel remodeling and muscle consumption in cigarette smoke-induced emphysema rats

**DOI:** 10.1186/s12890-019-0826-6

**Published:** 2019-06-06

**Authors:** Li Lin, Gang Hou, Dan Han, Yan Yin, Jian Kang, Qiuyue Wang

**Affiliations:** Institute of Respiratory Disease, The First Hospital of China Medical University, No. 155 Nanjing North Street, Shenyang, 110001 China

## Abstract

**Background:**

This study assessed the effects of ursolic acid (UA) on airway-vessel remodeling and muscle atrophy in cigarette smoke (CS)-induced emphysema rats and investigated potential underlying mechanisms.

**Methods:**

Emphysema was induced in a rat model with 3 months of CS exposure. Histology and immunohistochemistry (IHC) stains were used to assess airway-vessel remodeling and muscle atrophy-associated changes. Levels of cleaved-caspase3, 8-OHdG, and S100A4 were measured in airways and associated vessels to evaluate cell apoptosis, oxidant stress, epithelial-to-mesenchymal transition (EMT), and endothelial-to-mesenchymal transition (EndMT)-associated factors. Western blot and/or IHC analyses were performed to measure transforming growth factor-beta 1(TGF-β1)/Smad2.3, alpha-smooth muscle actin (α-SMA), and insulin-like growth factor 1 (IGF1) expression. We also gave cultured HBE and HUVEC cells Cigarette Smoke Extract (CSE) administration and UA intervention. Using Western blot method to measure TGF-β1/Smad2.3, α-SMA, S100A4, and IGF1 molecules expression.

**Results:**

UA decreased oxidant stress and cell apoptosis in airway and accompanying vascular walls of cigarette smoke-induced emphysema model rats. UA alleviated EMT, EndMT, changes associated with airway-vessel remodeling and muscle atrophy. The UA effects were associated with IGF1 and TGF-β1/Smad2.3 pathways.

**Conclusions:**

UA reduced EMT, EndMT, airway-vessel remodeling, and musculi soleus atrophy in CS-induced emphysema model rats at least partly through IGF1 and TGF-β1/Smad2.3 signaling pathways.

## Background

Chronic obstructive pulmonary disease (COPD) is a systemic disease characterized by persistent respiratory symptoms and airway limitation. It has high prevalence and associated mortality, with a prevalence among people 40 years of age or older of 10.1% worldwide and 13.7% in China [1, 2] and was estimated to be the third leading cause of death worldwide in 2030 [3]. The disease gives rise to enormous social and economic burdens [4, 5]. Nearly 90% of COPD cases are caused by Cigarette Smoke (CS) [6, 7]. Major pathological manifestations of COPD include chronic airway inflammation, airway-vessel remodeling [8], and emphysema [9]. Muscle atrophy is an important complication of COPD [10]. Although they are significant indicators of poor prognosis [8, 11–13], there are currently no effective interventions for airway-vessel remodeling and muscle consumption in COPD.

Airway-vessel remodeling is the main contributor to pulmonary dysfunction in COPD [14]. Potential mechanisms contributing to the airway-vessel remodeling of COPD include proliferation of airway epithelial cells, vascular endothelial cells, airway and vessel smooth muscle cells, fibroblast-myofibroblast transformation, epithelial-to-mesenchymal transition (EMT), and endothelial-to-mesenchymal transition (EndMT) [15–17]. Beyond the well-known airway remodeling processes associated with COPD [18], there has been a growing interest in vessel remodeling in COPD [19–22], for which the mechanism is not yet clear. The 2018 GOLD guide suggested that pulmonary microvascular blood flow was abnormal in smokers with even mild COPD. Meanwhile, patients with moderate or severe COPD often show pronounced pulmonary vascular remodeling, leading to pulmonary hypertension and pulmonary heart disease, which are directly related to patient prognosis. Patients with combined pulmonary fibrosis and emphysema, which are on the rise, are at increased risk of pulmonary hypertension and have a worse prognosis than patients with emphysema only, further indicating that vessel remodeling affects COPD progression [23].

CS activated cell oxidant stress and apoptosis can promote Transforming growth factor-beta 1(TGF-β1) secretion [24–27]. The TGF-β1/Smads signaling pathways are thought to mediate CS-induced airway-vessel remodeling in COPD [8, 28–31]. TGF-β1 is a multi-functional cytokine that regulates angiogenesis, extracellular matrix deposition, and fibroblast/myofibroblast trans-differentiation [29, 32–34]. Among its downstream pathways, TGF-β1/Smad2.3 signaling is strongly implicated in EMT and EndMT, which play key roles in COPD-associated airway-vessel remodeling [15, 28, 30].

Sarcopenia is an important complication of COPD and an indicator of poor prognosis of COPD patients [12, 35]. Exercise and glucocorticoids stimulate muscle recovery with variable efficacy, depending on the patient’s clinical condition and medical treatment [36]. Insulin-like growth factor 1(IGF1) is thought to play a key role in bronchial epithelial and muscle cell regeneration in COPD patients [36–39]. Thus, IGF1 intervention may contribute to treat COPD through effects on airway-vessel remodeling and muscle atrophy.

Ursolic acid (UA), a pentacyclic triterpenoid compound exits in many plants. It has anti-oxidant [40, 41], anti-inflammatory [42], anti-tumor [43], anti-apoptotic [44], and anti-fibrotic effects [45], all of which could support COPD treatment. In our previous experiment, we found UA intervention could alleviate CS induced emphysema in rats [46]. Researchers reported previously that UA is an antagonist of TGF-β1 [47], but whether UA exerts its effect through TGF-β1/Smads pathways remained unknown, especially in the context of COPD. UA was also reported to alleviate muscle consumption through the IGF1 pathway in an animal model of chronic kidney disease [48]. However, whether UA can alleviate CS-induced airway remodeling and muscle consumption in emphysema rats, and whether UA exerts its effects through TGF-β1/Smads and IGF1 pathways, remains to be established.

Therefore, we used CS induced rat emphysema model to assessed the effect of UA on EMT, EndMT, airway-vessel remodeling and muscle consumption and discuss the underlying mechanisms through TGF-β1/Smads pathways and IGF1 molecule. This study offered a new ademption for the treatment of clinical COPD patients.

## Methods

### Compounds and reagents

Antibodies against TGF-β1, 8-OHdG, α-SMA, S100A4, and IGF1 were obtained from Abcam Biotechnology Company (Cambridge, UK). Antibodies against Smad2, p-Smad2, Smad3, p-Smad3, and cleaved-caspase3 were purchased from Cell Signaling Technology (Denver, CO). UA was purchased from Wanxiang Hengyuan Biotechnology (Tianjin, China). Masson and Alcian blue-periodic acid Schiff (AB-PAS) kits were obtained from Nanjing Jiancheng biological engineering research institute (Nanjing, China). BCA kit was obtained from Pierce (Thermo-Scientific, Rockford, IL, USA), ECL chemiluminescence kit from Applygen (Beijing, China).

### Animals

Six-week-old male Wistar rats, weighing between 150 and 250 g, were bought from Chansheng Biotechnology Company (Liaoning, China). After two weeks of adaptation time, rats were randomized into one of five treatment groups (*N* = 10 each): Sham, CS, UA10, UA20, and UA40. UA rats were administrated 10 mg/kg, 20 mg/kg or 40 mg/kg body weight UA via gavage thirty minutes before the first CS exposure every day. Sham and CS rats were given vehicle instead. CS and UA rats were exposed to smoke of 16 filters removed 1R3F cigarettes for 30 min, two times a day, 6 days a week, for 3 months. Rats from the same group were placed five at a time into a glass chamber measuring 0.8 m × 0.6 m × 0.6 m, with a 2 cm × 2 cm spiracle on the top of the box. The time interval between the two exposures each day was 4 to 6 h. Rats in the Sham group were exposed to normal air. [46] The Animal Care and Use Ethics Committee of China Medical University approved this study.

### Cells culture and interventions

Human bronchial epithelial cells (HBEs) and human umbilical vein endothelial cells (HUVECs) were purchased from Peking University Cancer Institute (Beijing, China). Cells were cultured in RPMI-1640 culture medium (Hyclone, UT, USA) containing 10% fetal bovine serum (Hyclone, UT) in a 5% CO2 humidified cell incubator (Thermo Fisher Scientific, Inc., USA) at 37 °C. We treated 1 × 10^6^ cells with 10 μM/L UA 2 h prior to 1% cigarette smoke extract (CSE) [49] intervention. The concentrations we used were according to the CCK8 cytotoxicity testing (Dojido, Japan).

### Pathological materials

The left lungs were inflated and fixed using 4% phosphate- buffered formaldehyde (pH 7.40) at 25cmH2O pressure for 24 h. The musculi soleus muscles were fixed using 10% formaldehyde for 24 h. Then lungs and musculi soleus muscles embedded with paraffin. The paraffin-embedded sections were used for histopathological examination. Right lung tissues were frozen in liquid nitrogen for 5 min before storing at − 80 °C.

### Histopathology

We used hematoxylin and eosin (HE) staining to observe pathological changes to pulmonary airways and vessels. We measured and compared mean thickness of the airways and associated vessels in lung tissues. We observed and compared the pathological changes of musculi soleus using HE staining. To evaluate bronchial and vascular wall thickness, four sections that did not include cartilage but did include intact bronchial tracheal transections and concomitant vessels were randomly selected. For all bronchial sections, the ratio of minimum diameter to maximum diameter was> 0.5. Image Pro Plus 5.0 image analysis software (Media Cybernetics company, Maryland, American) was used to measure airway and vascular basement membrane perimeter (Pbm), airway wall area (total wall area [Wat]) and vascular wall area (total vascular area [Vat]). Wat/Pbm and Vat/Pbm values were calculated for each trachea and associated vessel, and the average value was used to compared airway and vascular wall thicknesses among groups.

### Masson staining

We used Masson staining to measure collagen deposition around the airways and vessels. Paraffin sections were dewaxed to water, then stained according to the Masson staining kit instructions (Nanjing Jiancheng Biotechnology, Nanjing, China). Areas of collagen deposition around airways and vessels were compared using the index wall area of collagen deposition (Wac)/Wat and vascular area of collagen deposition (Vac)/Vat measured using Image Pro Plus 5.0 image analysis software.

### AB-PAS staining

We used AB-PAS staining to count mucus-producing cells surrounding the airway. Paraffin sections were dewaxed to water, then stained according to the AB-PAS staining kit instructions (Nanjing Jiancheng Biotechnology, China).

### Immunohistochemistry (IHC)

Briefly, paraffin embedded tissues cut into 4-μm thick sections, and dewaxed, rehydrated. The slices were treated with H2O2 in methanol to inhibit endogenous peroxidase activity. Then antigen retrieval was performed using a microwave and 10-mM citrate buffer, pH 6. Slices were incubated with anti-8-OHdG, anti-cleaved caspase-3, anti-α-SMA, anti-TGF-β1, anti-p-Smad2, and anti-S100A4 antibodies overnight with the concentration of 1:500 at 4 °C. After washing, secondary antibodies were incubated room temperature for 1 h using the concentration of 1:1000. Then incubation with 3,3′-diaminobenzidine (DAB) and DAB Enhancer. One horizon in each quadrant of each section was assessed. Relative expression was compared using relative integrated optical density (IOD) surrounding the airway (IOD/Wat) and vessel (IOD/Vat), as measured using IPP 5.0 software. [46]

### Western blot analysis

Lung tissues, HBE cells and HUVEC cells lysates were prepared. Briefly, tissues and cells were lysed in an ice-cold lysis buffer (Roche Applied Science, Indianapolis, IN). Samples were then homogenized for 15 s at 4 °C, 4–5 times. Cell lysates were centrifuged at 12,000×*g* for 30 min at 4 °C to remove cellular debris. Protein concentration was determined using a BCA protein assay kit. Equal amounts of protein (20–60 μg) were separated on 8–10% sodium dodecyl sulfate-polyacrylamide gel electrophoresis gels and then transferred to PVDF membranes (Merck Millipore, Darmstadt, Germany), blocked and incubated with diluted primary antibodies overnight at 4 °C refrigerator. Blots were stripped and re-probed with anti-GAPDH antibody to demonstrate equal loading. Incubated with secondary antibody, the chemiluminescent signal was detected using the Super Enhanced Chemiluminescence Kit (Bio-Rad Laboratories, Shanghai, China). Band density was quantified using Quantity One software (Bio-Rad Laboratories, Shanghai, China). [46]

### Statistical analysis

SPSS13.0 software and Graph Pad Prism 5.0 software were used for statistical analysis. Kolmogorov Smirnov and Shapiro tests were used to assess normality, and all the data fit a Gaussian distribution. Data are presented as Mean ± Standard Deviation (SD). One-way analyses of variance (ANOVAs) were used to compare differences among groups. *p* < 0.05 was considerate to with statistical difference.

## Results

### UA decreased musculi soleus atrophy in CS-induced emphysema rats

**Fig. 1:**
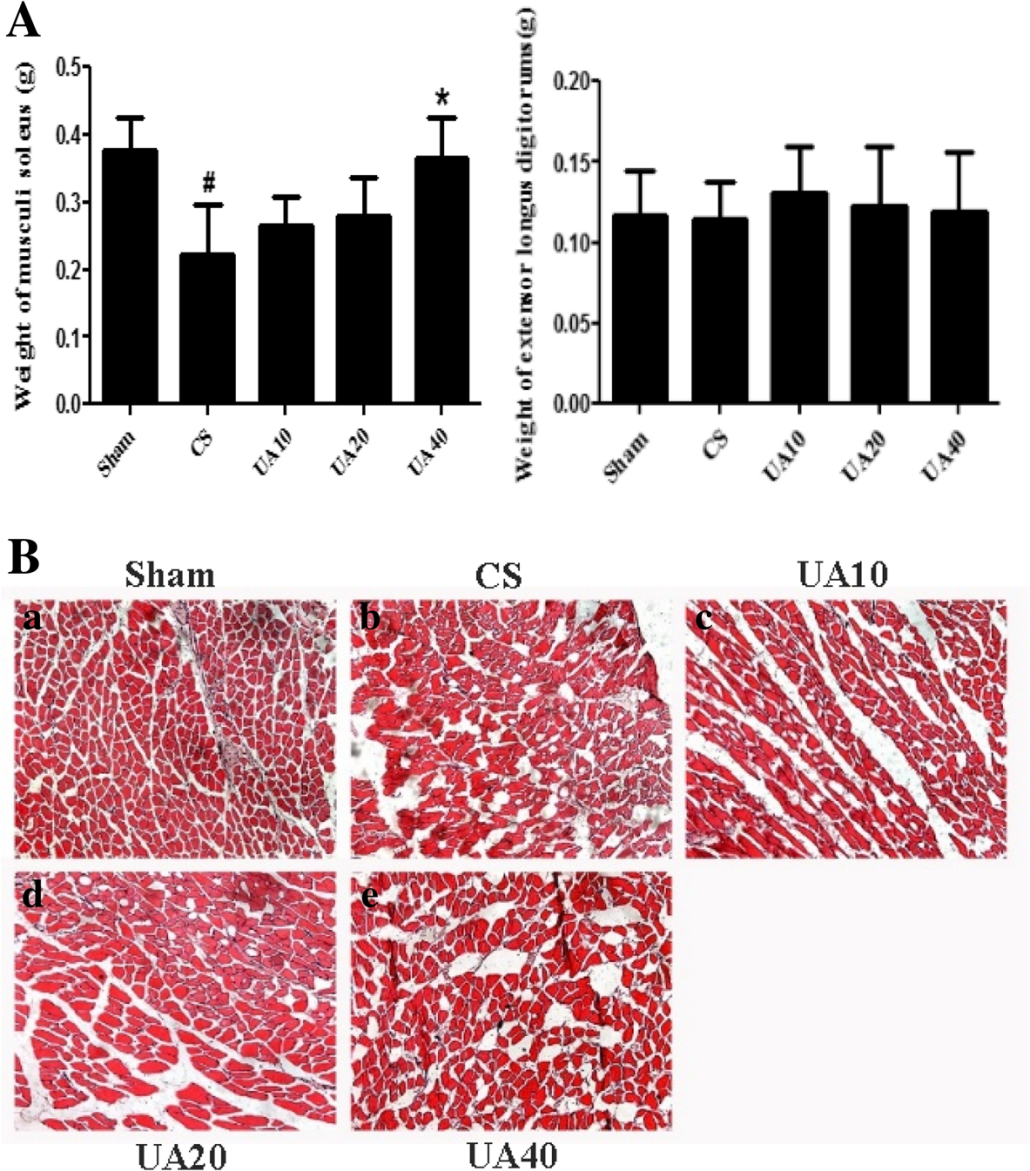
UA attenuate CS induced muscle atrophy in emphysema model rats. **A** Weights of musculi soleus and extensor digitorum longus. 40 mg/kg body weight UA administration attenuated CS induced musculi soleus loss. ^#^*p* < 0.05 vs. Sham; *p < 0.05 vs. CS. **B** Morphological changes of musculi soleus. HE staining of musculi soleus showed muscle atrophy associate changes in CS, and these changes were alleviated by UA intervention, especially in UA20 and UA40. Sham (a), CS (b), UA10 (c), UA20 (d), and UA40 (e). UA: ursolic acid; CS: cigarette smoke (group); UA10: 10 mg/kg body weight UA administration group; UA20: 20 mg/kg body weight UA administration group; UA40: 40 mg/kg body weight UA administration group

We had 10 rats in each group at the beginning of the experiment, but 2 rats in the CS group died during CS exposure. The remaining 48 rats survived and were used in subsequent experiments. Musculi soleus weights were significantly reduced in the CS group, relative to the Sham group, and 40 mg/kg body weight UA administration significantly attenuated this muscle mass loss. The same trend was not observed for extensor digitorum longus (Fig. 1A). Examination of HE stained sections revealed marked muscle atrophy with characteristic muscle cell crinkle, cell vacuolation, and structural disorder in the musculi soleus of CS-induced emphysema model rats, relative to the muscles of Sham rats. In UA groups, musculi soleus atrophy related changes such as muscle cell crinkle, vacuolation, muscle fiber disorder, and gap dilation were all improved in a concentration dependent manner. Myoarchitecture in UA20 and UA40 groups were similar to that of the Sham group (Fig. 1B).

### UA alleviated airway-vessel remodeling in CS-induced emphysema rat lungs

**Fig. 2:**
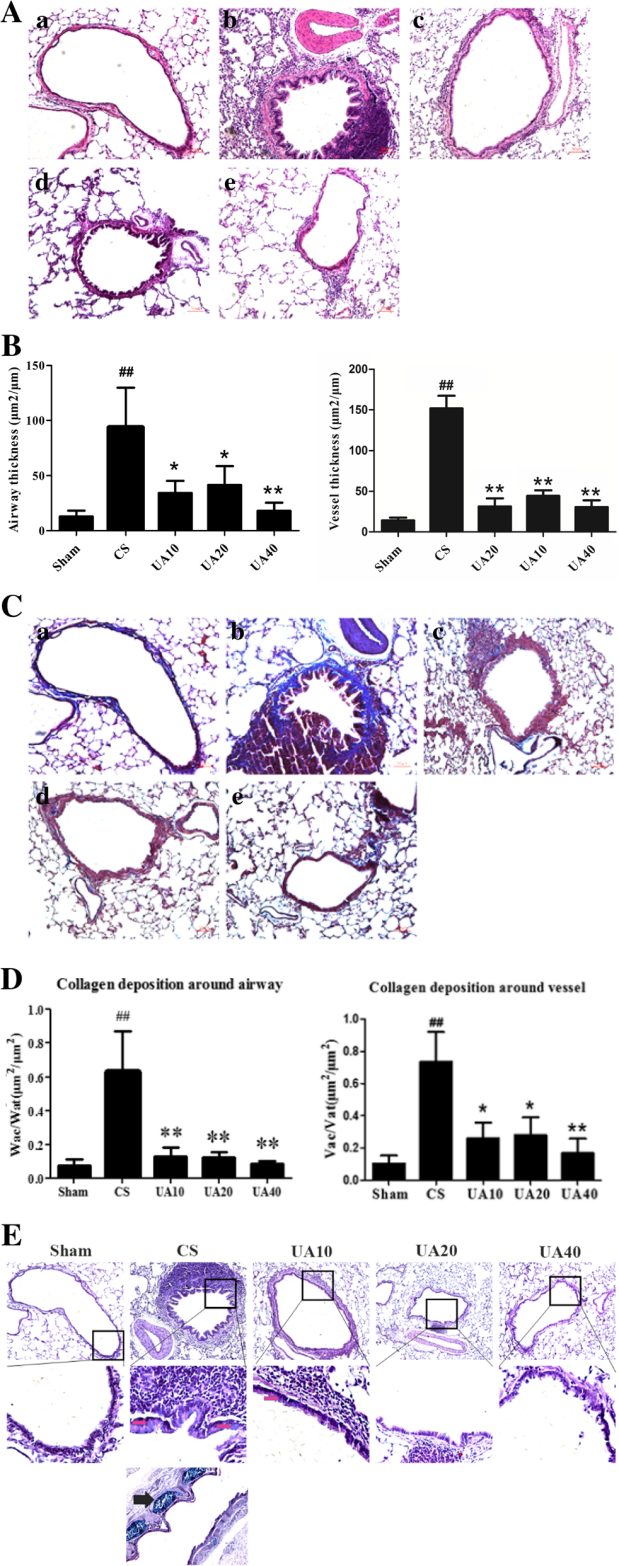
UA alleviate airway-vessel remodeling in emphysema model rats. **A** HE staining of lung sections showed that UA administration reduced the airway-vessel remodeling associated changes in CS-induced emphysema rats. Sham (a), CS (b), UA10 (c), UA20 (d), and UA40 (e). **B** Airway thickness (Wat/Pbm: μm^2^/μm) and vascular thickness (Vat/Pbm: μm^2^/μm). ^##^*p* < 0.01 vs. Sham; *p < 0.05, **p < 0.01 vs. CS. **C** Masson staining of lung sections showed that UA alleviated collagen deposition in tissue surrounding airways and accompanying vessels in lungs of emphysema rats. Sham (a), CS (b), UA10 (c), UA20 (d), and UA40 (e). **D** Collagen deposition in areas surrounding airways (Wac/Wat: μm^2^/μm^2^) and vessels (Vac/Vat: μm^2^/μm^2^). ^##^*p* < 0.01 vs. Sham; *p < 0.05, **p < 0.01 vs. CS. **E** PAS staining of lung sections showed that UA alleviated mucus secretion cell expression around airways in lungs of emphysema rats. Pink arrows show goblet cells. Black arrow shows mucous gland near the major airway. UA: ursolic acid; CS: cigarette smoke (group); UA10: 10 mg/kg body weight ursolic acid administration group; UA20: 20 mg/kg body weight ursolic acid administration group; UA40: 40 mg/kg body weight ursolic acid administration group; Pbm: airway or vessel basement membrane perimeter; Wat: airway wall area; Vat: vascular wall area; Wac: wall area of collagen deposition; Vac: vascular area of collagen deposition

HE stained sections revealed that the airways and associated vessels of the emphysema rats underwent airway-vessel remodeling, UA administration alleviated CS induced airway and vessel remodeling. The pathology changes observed in CS rats included proliferation and exfoliation of airway epithelial cells, airway basement membrane thickening, collagen deposition, airway contracture, infiltration of inflammation cells around airways, and vessel thickening. UA administration at all three concentrations alleviated these changes (Fig. 2A). We used airway and vessel thickness to quantify changes associated with airway-vessel remodeling. Compared with Sham group, the thickness of airways and accompanying vessels were remarkably increased with CS, and UA administration alleviated these changes at all three concentrations tested (Fig. 2B).

### Effects of UA on collagen deposition around airways and vessels caused by CS exposure

Masson staining showed that blue-dyed collagen deposition areas were significantly increased in airway and accompanying vessel walls of CS rats, compared with those in the Sham rats. Blue-dyed areas around airways and accompanying vessels were reduced significantly by all doses of UA administered (Fig. 2C). We used blue-stained areas in airway or vessels divided by the total areas of airway or vessels to compare the relative extent of collagen deposition. Proportion of collagen deposition areas to total airway or vessel areas are increased significantly in CS group compared with Sham. UA administration reduced the proportion of collagen deposition around airways and accompanying vessels (Fig. 2D).

### Effects of UA on airway mucus secretion cell expression caused by CS exposure

We used AB-PAS staining to assess mucus secreting cell expression surrounding the airways. Many large blue-dyed goblet cells were present in the epithelial layer in the airways of CS rats compared with Shams. UA decreased the size and number of goblet cells in epithelial layer of CS rats. We also noted metaplasia of large blue-dyed mucous glands in the large airway in the CS group (Fig. 2E).

### Effects of UA on airway and vessel smooth muscle cell proliferation caused by CS exposure

**Fig. 3:**
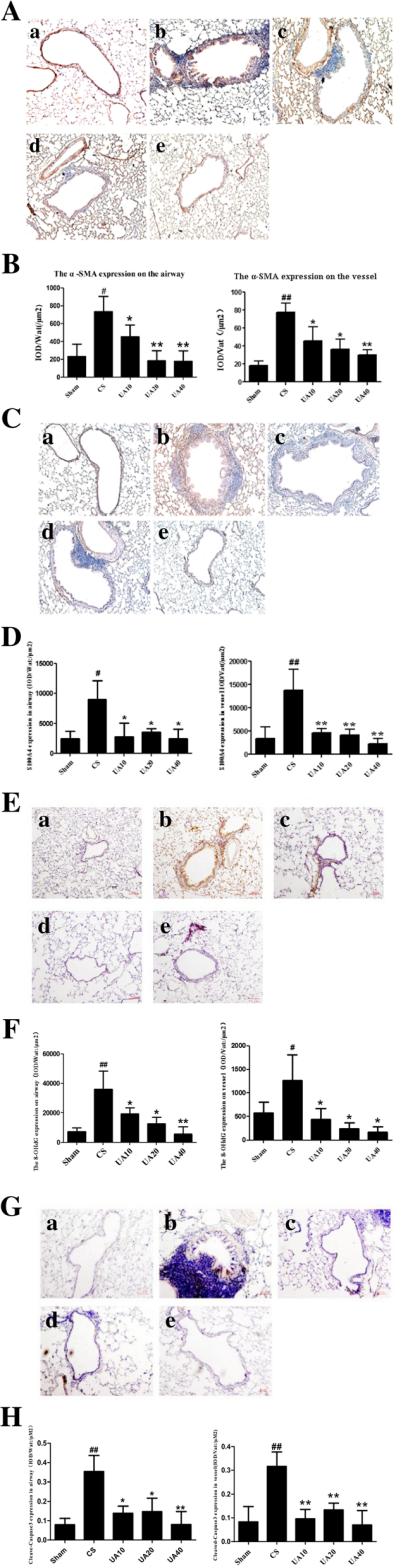
UA alleviate oxidant stress and cell apoptosis in airways and associated vessels, attenuated EMT/EndMT in CS-induced emphysema rats. **A** IHC staining of lung sections showed that UA reduced α-SMA expression in airways and vessels in emphysema rat lung, especially in the epithelial and endothelial layers. Sham (a), CS (b), UA10 (c), UA20 (d), and UA40 (e). **B** IHC IOD of α-SMA expression in airways and vessels. IOD/Wat: /μm^2^ and IOD/Vat: /μm^2^ data are shown. ^#^*p* < 0.05 ^##^*p* < 0.01 vs. Sham; *p < 0.05, **p < 0.01 vs. CS. **C** IHC staining of lung sections showed that UA reduced S100A4 expression in airway and vessel, especially in the epithelial and endothelial layers. Sham (a), CS (b), UA10 (c), UA20 (d), and UA40 (e). **D** IHC IOD of S100A4 expression in airways and vessels. IOD/Wat: /μm^2^ and IOD/Vat: /μm^2^ data are shown. ^#^*p* < 0.05, ^##^*p* < 0.01 vs. Sham; *p < 0.05, **p < 0.01 vs. CS. **E** IHC staining of lung sections showed that UA reduced 8-OHdG expression in airways and accompanying vessels in emphysema rat lungs, especially in the epithelial and endothelial layers. Sham (a), CS (b), UA10 (c), UA20 (d), and UA40 (e). **F** IHC IOD of 8-OHdG expression in airways and vessels. IOD/Wat: /μm^2^ and IOD/Vat: /μm^2^ data are shown. ^#^*p* < 0.05, ^##^*p* < 0.01 vs. Sham; *p < 0.05, **p < 0.01 vs. CS. **G** IHC staining of lung sections showed that UA reduced cleaved-caspase3 expression in airways and accompanying vessels in emphysema rat lungs, especially in the epithelial and endothelial layers. Sham (a), CS (b), UA10 (c), UA20 (d), and UA40 (e). **H** IHC IOD of cleaved-caspase3 expression in airways and vessels. IOD/Wat: /μm^2^ and IOD/Vat: /μm^2^ data are shown. ^##^*p* < 0.01 vs. Sham; *p < 0.05, **p < 0.01 vs. CS. UA: ursolic acid; CS: cigarette smoke (group); UA10: 10 mg/kg body weight UA administration group; UA20: 20 mg/kg body weight UA administration group; UA40: 40 mg/kg body weight UA administration; Pbm: airway or vessel basement membrane perimeter; Wat: airway wall area; Vat: vascular wall area; IHC: immunohistochemistry; IOD: integral optical density

To investigate smooth muscle cell proliferation, fibroblast to myofibroblast differentiation, EMT, and EndMT, we examined α-SMA expression in the area surrounding the airways and accompanying vessels using IHC. α-SMA expression around airways and vessels were significantly increased in the CS group compared with the Sham group, especially in the epithelial and endothelial layers. UA decreased α-SMA expression in the area surrounding airways and vessels, particularly in the epithelial and endothelial layers, at each dose of UA (Fig. 3A). The percentage of α-SMA staining areas per airway or vessel areas were significantly increased in CS rats compared with Sham rats, but UA administration decreased the percent of α-SMA staining area at all doses (Fig. 3B).

### Effects of UA on EMT, EndMT in airways and accompanying vessels caused by CS exposure

We used S100A4 expression to assess EMT and EndMT around airways and vessels. IHC analyses showed that S100A4 expression surrounding airways and accompanying vessels was significantly increased in the CS group compared with the Sham group. This increase was suppressed by UA, especially in the epithelial and endothelial layers (Fig. 3C). The percentage of S100A4 staining areas per airway or vessel areas were significantly increased in CS rats compared with Sham rats, and UA administration reversed this effect at all doses (Fig. 3D).

### Effects of UA on oxidant stress and cell apoptosis around airways and vessels caused by CS exposure

We used 8-OHdG and cleaved-caspase3 expressions to assess cell apoptosis around airways and vessels. IHC analyses showed that 8-OHdG and cleaved-caspase3 expression surrounding airways and vessels was significantly increased in the CS group compared with the Sham group. This increase was suppressed by UA, particularly in the epithelial and endothelial layers (Fig. 3E, G). The IOD/area values are consistent with the results above (Fig. 3F, H).

### Effects of UA on TGF-β1/Smad2.3 pathway and IGF1 expression caused by CS exposure

**Fig. 4:**
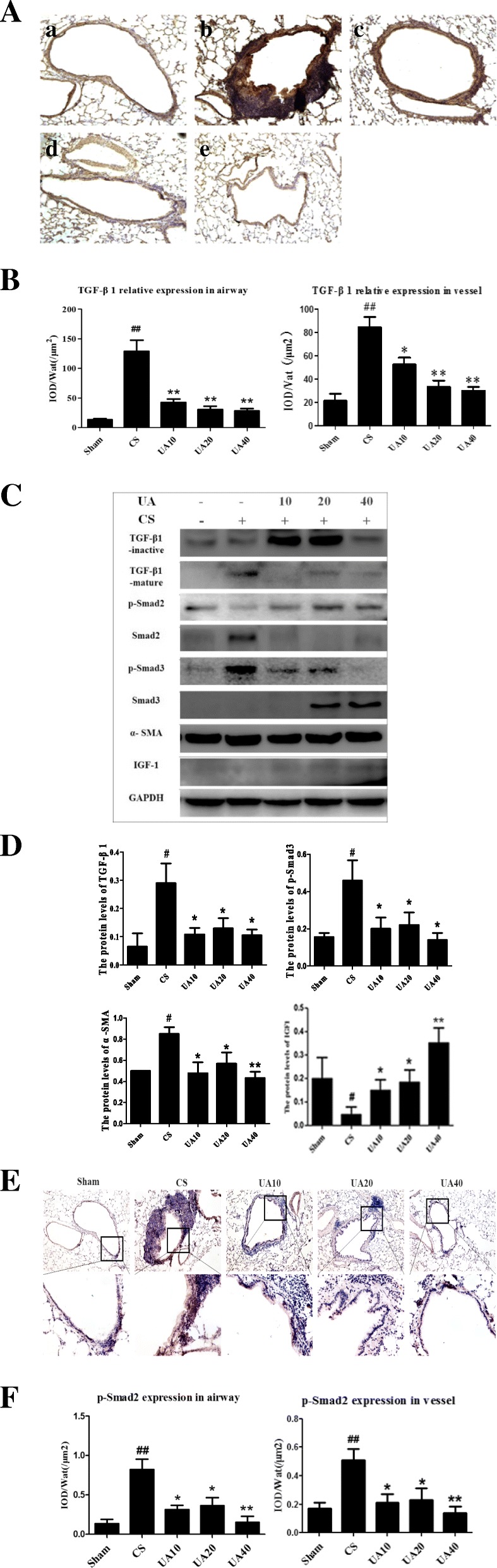
UA intervention alleviate TGF-β1/smad2.3 pathway molecules expression in airways and accompanying vessels of emphysema rats. **A** IHC staining of lung sections showed that UA reduced TGF-β1 expression in tissue surrounding airways and vessels in rat lungs. Sham (a), CS (b), UA10 (c), UA20 (d), and UA40 (e). **B** IHC IOD of TGF-β1 expression in airways and vessels. IOD/Wat and IOD/Vat: /μm^2^ data are shown. ^##^*p* < 0.01 vs. Sham; *p < 0.05, **p < 0.01 vs. CS. **C** Western blot analysis of TGF-β1/Smad2.3 pathway molecules and IGF1 expression in the lungs of emphysema rats. **D** Western blot IOD of TGF-β1/Smad2.3 pathway molecules and IGF expression in lungs. ^#^*p* < 0.05 vs. Sham; *p < 0.05, **p < 0.01 vs. CS. **E** IHC staining of lung sections showed that UA reduced p-Smad2 expression in tissue surrounding airways and vessels in rat lungs. Sham (a), CS (b), UA10 (c), UA20 (d), and UA40 (e). **F** IHC IOD of p-Smad2 expression in airways and vessels. IOD/Wat and IOD/Vat: /μm^2^ data are shown. ^##^*p* < 0.01 vs. Sham; *p < 0.05, **p < 0.01 vs. CS. UA: ursolic acid; CS: cigarette smoke (group); UA10: 10 mg/kg body weight UA administration group; UA20: 20 mg/kg body weight UA administration group; UA40: 40 mg/kg body weight UA administration; Pbm: airway or vessel basement membrane perimeter; Wat: airway wall area; Vat: vascular wall area; IHC: immunohistochemistry; IOD: integral optical density

IHC analysis further showed markedly upregulated TGF-β1 expression in airways and accompanying vessels, while in UA groups, TGF-β1 expression was downregulated in all of the UA groups, especially in the epithelial and endothelial layers (Fig. 4A). These IOD/area values are consistent with the results above (Fig. 4B). Western blot analysis of TGF-β1, p-Smad2, p-Smad3, Smad2, Smad3, α-SMA, and IGF1 expression showed that the activated forms of TGF-β1, p-Smad3, and α-SMA were upregulated in rat lung in the CS group compared with the Sham group. In the UA groups, active forms expression of TGF-β1, p-Smad3, and α-SMA was down-regulated. IGF1 expression was down-regulated in the CS group compared with the Sham group. UA administration upregulated IGF1 expression in rat lung (Fig. 4C and D). Contrary to expectations, the p-Smad2 expression seemed to be down-regulated in the CS group but upregulated in the UA groups. Thus, we used IHC to assess p-smad2 expression around airways and accompanying vessels. It revealed markedly upregulated p-Smad2 expression in airways and accompanying vessels, but not pulmonary parenchyma regions, downregulated p-Smad2 expression in airways and accompanying vessels after UA treatment, which is inconsistent with the IOD results (Fig. 4E and F).

### Effects of UA on TGF-β1/Smad2.3 pathway molecules expression in HBE and HUVEC cells after CSE administration

**Fig. 5:**
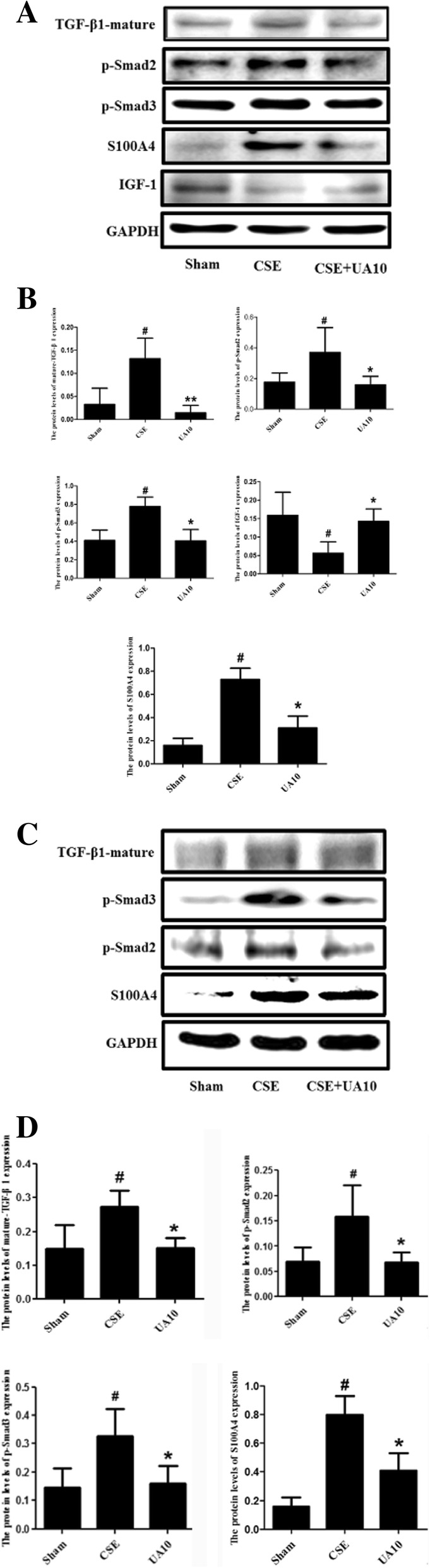
UA alleviated CSE-induced TGF-β1/Smad2.3 pathway proteins and S100A4 expression in HBEs and HUVECs in vitro. **A** Western blot analyses showed UA attenuated CSE-induced TGF-β1/Smad2.3 pathway protein and S100A4 expression in HBEs. **B** IOD of TGF-β1/smad2.3 pathway, IGF1, and S100A4 expression in HBEs ^#^*p* < 0.05 vs. Sham; *p < 0.05 vs. CSE. **C** Western blot analyses showed that UA alleviated CSE-induced TGF-β1/Smad2.3 and S100A4 protein expression in HUVECs. **D** IOD of TGF-β1/Smad2.3 pathway proteins and S100A4 expression in HUVECs. ^#^*p* < 0.05 vs. Sham; *p < 0.05 vs. CSE. UA: ursolic acid; CSE: cigarette smoke extract (group); HBE: Human Bronchial Epithelial cells; HUVEC: Human Umbilical Vein Endothelial Cells; IOD: integral optical density.

Western blot analysis showed upregulated expression of activated forms of TGF-β1, p-Smad2, p-Smad3, and S100A4 in HBEs and HUVECs after CSE administration compared with vehicle control cells, that could be attenuated by UA treatment. CSE administration downregulated IGF1 expression in HBE cells, UA alleviated the IGF1 down-regulation (Fig. 5A-D).

## Discussion

The present study showed that UA treatment alleviated EMT, EndMT, airway-vessel remodeling, and muscle atrophy-associated lesions in a rat model of CS-induced emphysema. We further demonstrated that UA exerts its effects through mechanisms that involve upregulation of IGF-1 and inhibition of the TGF-β1/Smad2,3 pathway.

Similar to EMT, of which it is a special type, EndMT refers to the process of under-stimulated endothelial cells taking on a mesenchymal cell phenotype. EndMT is characterized by decreased cellular connectivity, reduced expression of endothelial markers (e.g., CD31 and cadherin), increased expression of mesenchymal markers (e.g., α-SMA and vimentin), and signs of cellular invasion and migration. Recent observations of upregulated expression of S100A4 in the vascular walls of COPD patients suggest that EndMT may be involved in COPD pulmonary vascular remodeling [16, 17], though the mechanism underlying these changes is unclear.

We found in this study, 3 months of CS exposure induced significant airway-vascular remodeling in rat lungs. Pathological manifestations included increased thickness of the airways and accompanying vessels, as well as collagen deposition in these areas. Remodeling increased the size and quantity of goblet cells in the epithelial layer of the airway, as well as mucinous gland metaplasia in areas surrounding the central airway. Remodeling also involved increased expression of α-SMA and S100A4 in airways and accompanying vessels. Expression of S100A4 is specific index for EMT and EndMT.

3 months of CS exposure also increased TGF-β1 expression in airway and vessel walls as well as whole lung of rats, and its downstream p-Smad3 expression. However, western blot analyses showed decreased p-Smad2 expression in whole lung of rats, contrasting with previous findings [50]. We sought to explain this discrepancy.

In a previous study of Smad2 activation in the lung tissues of COPD patients, Lepparanta and colleagues found reduced Smad2 activation in alveoli and increased Smad2 activation in thickened bronchial tissues. Down-regulation of p-Smad2 expression in emphysema rat lungs may due to imbalanced expression of Smad2 in pulmonary parenchyma and airway-vessels [51]. Our IHC analysis of p-Smad2 expression in airways and vessels showed upregulation of p-Smad2 in airway and vessel walls of CS-induced emphysema model rats. We also observed higher levels of TGF-β1/Smad2,3 pathway constituents and increased S100A4 expression in CSE-exposed HBEs and HUVECs.

UA, a compound that comprises three terpenoids found in plants, has a wide range of effects, which may inhibit the occurrence and development of COPD. Previously, we found that UA administration significantly alleviated body weight loss, oxidative stress, and cell apoptosis in lung tissue of CS induced emphysema rats. UA exerted its effects through the unfolded protein response (UPR) PERK and Nrf2 pathways [46]. We also found previously that IRE1 pathway, but not ATF6 pathway, signaling was upregulated this model. In this study, we found that UA alleviated EMT, EndMT, airway-vessel remodeling, and muscular atrophy in the same model, and that it does so partly through TGF-β/Smad2.3 and IGF-1 signaling pathways. These results suggest that UA could exert dual effects in rats with CS-induced emphysema.

The UPR of endoplasmic reticulum stress (ERS) has been described involving in EMT in other disease processes [52–55]. It is not yet known whether a similar activation of the UPR occurs during EMT of airway and alveolar epithelial cells in COPD. Recently, Liang and colleagues proposed that ERS induced by advanced oxidation protein products may be involved in glomerular endothelial cell EndMT, leading to the development of diabetic nephropathy [56]. Meanwhile, Ying and colleagues proposed that ERS-induced EndMT may occur through the Src pathway in HUVECs [57]. It remains to be established whether activation of the unfolded protein response plays a role in EMT/EndMT during COPD-associated airway-vessel remodeling, and if so, which pathways are most critical. Furthermore, it will be of interest to determine whether UA treatments that alleviate airway-vessel remodeling affect the unfolded protein response in association with endoplasmic reticulum stress.

However, our findings are insufficient to identify the exact mechanism underlying the effects of UA on CS-induced airway-vessel remodeling. The nature of the link between EndMT and COPD has yet to be clarified [16, 17]. These observations are important and warrant further studies.

## Conclusion

Adding to our previous study showing that UA can alleviate CS-induced emphysema in rats via attenuation of oxidative stress and cell apoptosis, here we show that UA can also alleviate CS-induced EMT, EndMT, airway-vessel remodeling, and muscle atrophy. As a compound that occurs naturally in plants, and has already been used for clinical trials in solid tumors, UA offers much promise as an intervention for the pathogenesis, symptoms, and complications of COPD.

## Abbreviations

EMT: epithelial-to-mesenchymal transition
EndMT: endothelial-to-mesenchymal transition
IGF1: insulin-like growth factor 1
Pbm: basement membrane perimeter
TGF-β1: transforming growth factor-beta 1
UA: ursolic acid
Vac: vascular area of collagen deposition
Vat: total vascular area
Wac: wall area of collagen deposition
Wat: total wall area
